# Yoga as a Therapeutic Intervention in Cancer Care: An Umbrella Review of Systematic Reviews and Meta-Analyses

**DOI:** 10.7759/cureus.62668

**Published:** 2024-06-19

**Authors:** Selvaraj Giridharan, Jawaher Ansari, Nandan M Shanbhag, Khalid Balaraj

**Affiliations:** 1 Oncology, Tawam Hospital, Al Ain, ARE; 2 Oncology/Radiation Oncology/Palliative Care, Tawam Hospital, Al Ain, ARE; 3 Medicine and Health Sciences, United Arab Emirates University, Al Ain, ARE; 4 Oncology/Radiation Oncology, Tawam Hospital, Al Ain, ARE

**Keywords:** integrative oncology, umbrella review, systematic review, quality of life, psychosocial health, cancer care, yoga

## Abstract

The therapeutic efficacy of yoga in cancer care has increasingly attracted attention due to the imperative to address the physical and psychosocial obstacles encountered by cancer patients. Despite previous research presenting conflicting findings on the effectiveness of yoga, there is a need for a comprehensive review to consolidate existing evidence and identify commonalities across studies. An umbrella review was undertaken to aggregate and analyse findings from multiple systematic reviews and meta-analyses on the role of yoga in cancer care. Relevant literature was identified through searches on the Web of Science, Cochrane Library, PubMed, and Scopus databases, using a combination of MeSH (Medical Subject Headings) terms and free-text terms with Boolean operators. The quality of the included reviews was evaluated using the AMSTAR-2 tool to ensure the reliability and validity of the discussed findings.

The outcomes revealed a predominance of favourable results associated with yoga interventions, particularly in enhancing psychosocial well-being and the quality of life among cancer patients. Consistent reports indicated significant reductions in symptoms such as anxiety, depression, and stress, as well as enhancements in physical outcomes such as fatigue and sleep quality. However, variations in the efficacy of yoga were observed and were dependent on the type of intervention, patient adherence, and comparative analyses with other forms of exercise. While the benefits were substantial in the short term, they did not uniformly surpass those of other therapeutic exercises in the medium term.

Despite yoga demonstrating significant immediate benefits in managing both the physical and psychological symptoms associated with cancer, the variability in its long-term and comparative effectiveness suggests the necessity for personalised approaches. The findings emphasise the importance of considering individual patient needs and treatment contexts when integrating yoga into cancer care protocols. Future research should focus on identifying the optimal conditions under which yoga is most beneficial to tailor interventions for enhanced therapeutic efficacy.

## Introduction and background

Cancer continues to pose significant health challenges globally, profoundly impacting morbidity and mortality rates [1]. Advances in medical treatments have significantly increased the life expectancy of many cancer patients, leading to enhanced survival rates across various cancer types [2]. However, these improvements have resulted in a growing population of long-term cancer survivors who face numerous psychological and physical challenges.

The experience of cancer and its treatments can significantly impact survivors' psychological well-being, leading to increased levels of distress [3]. This psychological distress can adversely affect bio-behavioural responses, harm immune health and function, and contribute to immunological ageing [4]. Addressing these multifaceted issues is crucial for enhancing the quality of life (QOL) of cancer patients, highlighting the need for comprehensive care strategies that incorporate interventions such as yoga to manage the morbidities associated with cancer and its treatment.

Yoga, a scientific practice that originated in India over 5,000 years ago, has gained prominence for its potential benefits in managing treatment-related symptoms and improving health outcomes in cancer patients. Yoga is not a religion or belief system but a science encompassing various practices and techniques [5]. Although the exact mechanisms through which yoga supports physical and psychological improvement are not entirely understood, scientific evidence suggests it down-regulates the hypothalamic-pituitary-adrenal axis and the sympathetic nervous system.

Yoga involves physical postures (asanas), breathing exercises (pranayama), meditation, and ethical principles, creating a harmonious balance between the body, mind, and soul. Yoga is recognised for enhancing bodily systems, improving posture, regulating sleep, and reducing stress and anxiety, among other benefits [6]. It has positively impacted various health conditions, including neurological diseases, heart and lung diseases, and chronic pain [7-10]. Its holistic nature, addressing physical, emotional, and spiritual dimensions, makes it particularly relevant for individuals navigating the complexities of cancer treatment [11-14].

However, studies on yoga's effectiveness often produce inconsistent and contradictory results due to variations in study design, types of yoga interventions, and patient populations. This has led to a fragmented understanding of the extent to which yoga benefits cancer patients [15,16]. Therefore, there is a critical need for a comprehensive synthesis of the evidence to provide a more definitive and generalised conclusion about yoga's role in cancer care.

Consequently, there is a critical need for a comprehensive synthesis of the evidence that can provide a more definitive and generalised conclusion about the role of yoga in cancer care. The aim of this umbrella review, therefore, is to explore the aggregated evidence from systematic reviews and meta-analyses regarding the efficacy of yoga in managing cancer-related symptoms and enhancing life quality, thereby informing healthcare professionals, patients, and policymakers about the viability of incorporating yoga into standard cancer care protocols.

## Review

Methodology

For this umbrella review, the Preferred Reporting Items for Systematic Reviews and Meta-Analyses (PRISMA) guidelines [17] were adhered to to ensure clarity, transparency, and reproducibility of the research process. The PECO (Population, Exposure, Comparator, Outcome) protocol was defined as follows for this umbrella review: *Population *(*P*) was the population of interest, which comprised individuals of all ages diagnosed with cancer, regardless of stage or treatment phase; *Exposure *(*E*) was the exposure of interest, which was yoga interventions. These interventions could include any form of yoga practice, such as Hatha, Vinyasa, or restorative yoga, and were considered regardless of their duration, frequency, or intensity; *Comparator *(*C*) where the comparators did not include any intervention, usual care, or other non-yoga interventions used as control conditions in the original studies included in the systematic reviews and/or meta-analyses; and *Outcome *(*O*)where the outcomes of interest were cancer-related symptoms, psychological outcomes, QOL, and any adverse events associated with yoga interventions. Specific outcomes included, but were not limited to, fatigue, pain, sleep disturbances, stress, anxiety, depression, physical functioning, and overall survival rates where available.

The inclusion and exclusion criteria utilised in this review are mentioned in Table 1.

**Table 1:** The inclusion and exclusion criteria utilised across the review

Criteria	Inclusion	Exclusion
Study type	Systematic reviews and meta-analyses that evaluated the effects of yoga interventions on cancer patients.	Systematic reviews that did not include meta-analyses, literature reviews, commentaries, case reports, case series, and primary research studies.
Participants	Studies involving participants of any age diagnosed with any cancer.	Studies focusing on populations without a cancer diagnosis.
Interventions	Studies that included any form of yoga as an intervention.	Studies examining interventions not specifically involving yoga (e.g., other exercises or meditation).
Comparators	Comparisons against no intervention, usual care, or other non-yoga interventions.	Studies lacking a comparator group.
Outcomes	Outcomes related to physical symptoms, psychological well-being, quality of life, and adverse events associated with yoga.	Studies do not report specific outcomes relevant to the review's objectives.
Publication	No limitations were placed.	
Time frame		

Database Search Protocol

The search was conducted across four major databases: Web of Science, Cochrane Library, PubMed, and Scopus. Each database was queried using a combination of MeSH (Medical Subject Headings) terms and free-text terms with appropriate Boolean operators to ensure a broad yet specific capture of relevant literature, as shown in Table 2.

**Table 2:** Search strings utilised across the databases MeSH: Medical Subject Headings; TS: Topic Search; tiabkw: Title, Abstract, Keywords; TITLE-ABS-KEY: Title, Abstract, Keywords

Database	Search String
PubMed	(("Yoga"[MeSH Terms]) AND ("Neoplasms"[MeSH Terms] OR "Cancer"[Title/Abstract])) AND ("systematic review"[Title/Abstract] OR "meta-analysis"[Title/Abstract])
Web of Science	TS=(yoga) AND TS=(cancer OR neoplasm) AND TS=(systematic review OR meta-analysis) AND (SU=Oncology OR SU=Integrative Medicine)
Cochrane Library	((yoga:ti,ab,kw OR yogic:ti,ab,kw) AND (cancer:ti,ab,kw OR neoplasm*:ti,ab,kw OR oncolog*:ti,ab,kw) AND (systematic review:ti,ab,kw OR meta-analysis:ti,ab,kw))
Scopus	((TITLE-ABS-KEY (yoga) AND TITLE-ABS-KEY (cancer OR neoplasm)) AND (TITLE-ABS-KEY (systematic review OR meta-analysis)) AND (LIMIT-TO (SUBJAREA, "MEDI") OR LIMIT-TO (SUBJAREA, "HEAL")))

Variable Extraction Protocol

Two independent reviewers were tasked with extracting data using a standardised data extraction form that was developed and piloted prior to the commencement of the review process. Any reviewer discrepancies were resolved through discussion or consulting a third reviewer to reach a consensus. The data items selected for extraction were carefully chosen to provide comprehensive insights into the nature of the yoga interventions examined and their impacts on cancer patients. The following information was extracted from each included systematic review or meta-analysis: (1) general information*:* publication year, authors, geographical location of the studies included in the review, and journal of publication; (2) study characteristics: study design, total number of studies included, total participant numbers, types of cancer addressed, and cancer stages; (3)​​​​​​​ yoga interventions: types of yoga practised (e.g., Hatha, Vinyasa), duration of the yoga sessions, frequency and total duration of the intervention, settings in which the interventions were conducted (e.g., clinical settings, community centres), and qualifications of the yoga instructors; (4) comparators: types of control interventions used, such as usual care, no intervention, or alternative therapies; (5)​​​​​​​ outcomes: primary and secondary outcomes were measured, including physical symptoms (e.g., pain, fatigue), psychological outcomes (e.g., anxiety, depression), QOL, and any reported adverse events; and (6) ​​​​​​​results: main findings related to the effectiveness of yoga interventions on specified outcomes, including effect sizes and statistical significance where applicable.

Bias Assessment Protocol

For our review, the assessment of potential biases in the included systematic reviews and meta-analyses was conducted using the AMSTAR-2 (A Measurement Tool to Assess Systematic Reviews-2) tool [18], an advanced methodological quality assessment tool specifically designed for this purpose. The tool evaluates various aspects, including protocol registration, comprehensive literature search, explanation for study selection, risk of bias in included studies, appropriateness of meta-analytical methods, consideration of risk of bias, and assessment of publication bias. Each included review was evaluated against these criteria to ensure methodological rigour and transparency.

Results

**Figure 1:**
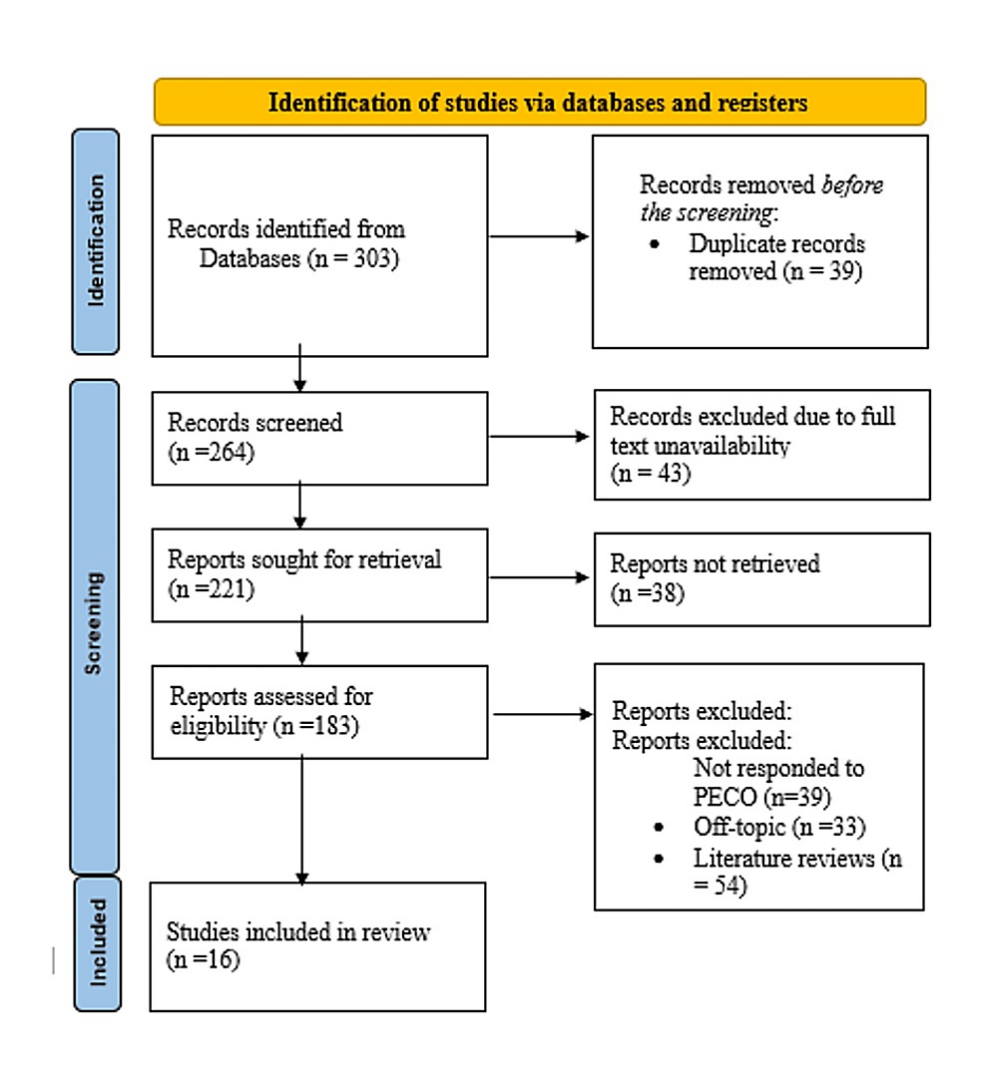
Summarised search strategy (Preferred Reporting Items for Systematic Reviews and Meta-Analyses flow diagram)

During the initial stage of the research selection process, 303 entries were found in all the evaluated databases (Figure 1).

After removing 39 duplicate entries, 264 records remained for review. A total of 43 items were inaccessible, so an attempt was made to obtain 221 reports. Of these, 38 records could not be accessed for further examination. The remaining 183 reports were thoroughly assessed for eligibility. Of these, 37 studies were off-topic, 54 were literature reviews, and 41 were scoping reviews that did not adhere to the PICO guidelines. After applying the exclusion criteria, 16 systematic reviews and meta-analyses [19-34] were deemed suitable for inclusion in the evaluation. In this section, we provide a detailed examination of these selected studies, which are also summarised in Table 3.

**Table 3:** Meta-analyses included in our investigation and their observed inferences SMD: Standardised Mean Difference; 95% CI: 95% Confidence Interval; I²: I-squared; MD: Mean Difference; WMD: Weighted Mean Difference; TNF-α: Tumour Necrosis Factor-alpha; IL-6: Interleukin-6; CRF: Cancer-Related Fatigue; 6MWT: Six-Minute Walk Test; QOL: Quality of Life

Study ID	Pain Assessment	Sleep Quality Assessment	Fatigue	Exercise Capacity	Cytokine Levels	Anxiety and Depression	Quality of Life	Overall Inference
Buffart et al. [19]	Large effect in pain reduction for breast cancer	Mixed: significant improvements in lymphoma but not in breast cancer	Moderate improvement in fatigue	Not reported	Not reported	Significant reductions in distress, anxiety, and depression	Significant improvements in functional well-being and quality of life	Beneficial effects on physical and psychosocial outcomes; adherence and tailored interventions crucial
Cramer et al. [20]	Not reported	Significant short-term improvement: SMD = -0.25, 95% CI (-0.40 to -0.09), I² = 0%	Short-term: significant benefit: SMD = -0.48, 95% CI (-0.75 to -0.20), I² = 72% Medium-term: no overall effect: SMD = -0.04, 95% CI (-0.36 to 0.29), I² = 0%	No data	Not reported	Short-term: no significant effects on depression or anxiety with very low to low-quality evidence due to inconsistency and imprecision. Vs. Psychological Interventions: Significant reduction in depression: SMD = -2.29, 95% CI (-3.97 to -0.61), I² = 96% and anxiety: SMD = -2.21, 95% CI (-3.90 to -0.52), I² = 95%	Yoga vs. no therapy: short-term significant improvement: SMD = 0.22, 95% CI (0.04 to 0.40), I² = 19% Yoga vs. exercise: no significant differences: SMD = -0.04, 95% CI (-0.30 to 0.23), I² = 6%	Yoga provides significant short-term benefits in quality of life, fatigue, and sleep quality but shows no significant medium-term effects or consistent superiority over exercise
Dong et al. [21]	Not reported	Not reported	Fatigue: Post-treatment: SMD = -0.80; Intra-treatment: SMD = -0.25; Hatha Yoga: SMD = 0.35; Iyengar Yoga: SMD = -0.17 to -0.98; Supervised classes: SMD = -0.92	Not reported	Not reported	Not reported	Not reported	Yoga significantly reduces fatigue post-treatment; effectiveness varies by yoga type and intervention form
El-Hashimi et al. [22]	Not reported	Not reported	Minimal impact; d = -0.14 to 0.50, often not significant	Not reported	Not reported	Varying effects; Significant in some studies (e.g., d = -1.80 for depression)	Immediate slight improvement not maintained long-term; d = 0.14 initial, fading over time	Initial slight psychosocial benefits not persistent; effectiveness highly contextual
Gonzalez et al. [23]	Not reported	Not reported	Not reported	Not reported	Not reported	Significant reduction in anxiety and depression symptoms; medium effect size for depression (g = -0.553, 95% CI = -0.781 to -0.325) and anxiety (g = -0.554)	Not reported	Yoga effectively reduces anxiety and depression among various cancer patients, with outcomes influenced by session frequency and treatment phase
Hou et al. [24]	Not reported	Significant improvement (MD = -3.86)	Significant reduction (SMD = -0.51)	Not reported	Not reported	Significant reduction in both anxiety (SMD = -0.93) and depression (SMD = -1.23)	Substantial improvement (MD = -11.20)	Yoga significantly reduces CRF, improves sleep, reduces anxiety and depression, and enhances overall QOL, with stable results despite heterogeneity
Hsueh et al. [25]	Significant reduction in pain severity (SMD = -0.38, 95% CI: -0.74, -0.02)	Significant improvement noted (WMD = -0.99, 95% CI: -1.95, -0.04)	Significant reduction in fatigue levels (SMD = -0.99, 95% CI: -1.56, -0.43)	Not specifically assessed	Not specifically assessed	Significant reductions in anxiety (SMD: –1.35) and depression (SMD: –0.98)	Significant improvements in social, emotional, and functional well-being	Yoga interventions effectively reduce stress, fatigue, and pain and improve sleep quality and psychosocial aspects of QOL in cancer patients
Lin et al. [26]	Not assessed	Not assessed	SMD = -0.15, 95% CI (N/A), p=0.24	Not assessed	Not assessed	Anxiety: SMD = -0.76, p=0.009 Depression: SMD = -0.95, p=0.002	SMD = -0.29, p=0.06	Significant benefits in reducing anxiety, depression, and stress, positive QOL trend
Niu et al. [27]	No effect initially, significant upon sensitivity analysis (n=89, SMD=-0.58)	Significant improvements at 4 and 8 weeks, no change at 12 weeks	Mixed results, significant at 10 weeks (n=180, SMD=-0.63)	There was no improvement in 6MWT; various other physical improvements were noted	Mixed outcomes for TNF-α, IL-6, significant decreases; variable results for other cytokines	Significant reductions at 8 and 12 weeks	Mixed results, significant improvements in global health and some symptoms	Yoga shows variable benefits across different health outcomes, significant in QOL, anxiety, depression reduction
O'Neill et al. [28]	Not reported	Not reported	CRF: Yoga vs. non-active: SMD = -0.30; Yoga vs. active: SMD = -0.17	Not reported	Not reported	Not reported	QOL: Yoga vs. non-active: SMD = 0.27; Yoga vs. active: SMD = -0.04	Yoga shows mixed effects on CRF and QOL, dependent on comparator type and measurement scales
Pan et al. [29]	No significant relief; SMD: -0.09, 95% CI: -0.64, 0.46	No significant improvement; SMD: -0.19, 95% CI: -0.39, 0.00	No significant relief; SMD: -0.22, 95% CI: -0.53, -0.09	Not reported	Not reported	Significant reduction in anxiety (SMD: -0.98, 95% CI: -1.38, -0.57) and depression (SMD: -0.17, 95% CI: -0.32, -0.01)	Significant improvement; SMD: 0.85, 95% CI: 0.37, 1.34	Significant improvements in anxiety, depression, and quality of life; mixed outcomes in physical symptoms
Song et al. [30]	Not specifically assessed	Not specifically assessed	Significant reduction in CRF, particularly with ≥150 minutes/week of yoga (SMD = –0.96, 95% CI: –1.52, –0.4)	Not specifically assessed	Not specifically assessed	Not specifically assessed	Not specifically assessed	Yoga significantly alleviates CRF, especially in breast cancer patients, with varying adherence linked to intervention duration and supervision
Tang et al. [31]	Not reported	Sleep: SMD = -0.42; I² = 54.11%	Not reported	Not reported	Not reported	Not reported	Not reported	Significant improvement in sleep disturbances; effects varied by adherence and measurement methods
Yi et al. [32]	Not assessed	SMD = -0.34, 95% CI (-0.55, -0.12), p=0.003	SMD = -0.62, 95% CI (-1.17, -0.07), p=0.03	Not assessed	Not assessed	SMD = -0.50, 95% CI (-0.70, -0.31), p<0.000	SMD = 0.72, 95% CI (-0.12, 1.56), p=0.09	Significant short-term benefits in managing symptoms, effects may not persist over time
Zhang et al. [33]	Not assessed	MD = -0.44, 95% CI (-2.54, 1.66), p=0.68	SMD = 0.11, 95% CI (-0.12, 0.35), p=0.35	Not assessed	Not assessed	Anxiety: SMD = -0.24, 95% CI (-0.54, 0.06), p=0.11 Depression: MD = -4.12, 95% CI (-13.05, 4.81), p=0.37	SMD = 0.27, 95% CI (0.02, 0.52), p=0.03	Significant benefits in stress reduction and QOL, no major benefits in other areas
Zhu et al. [34]	Not reported	Significant improvement in sleep quality with yoga, especially meditation-focused, 2-3 times per week for 6-8 weeks (SMD = -0.40, 95% CI: -0.71, -0.09, P = 0.01)	Not reported	Not reported	Not reported	Not reported	Not reported	Yoga significantly improves sleep quality in breast cancer patients; its effectiveness varies by type, frequency, and duration

Observed Bias

**Figure 2:**
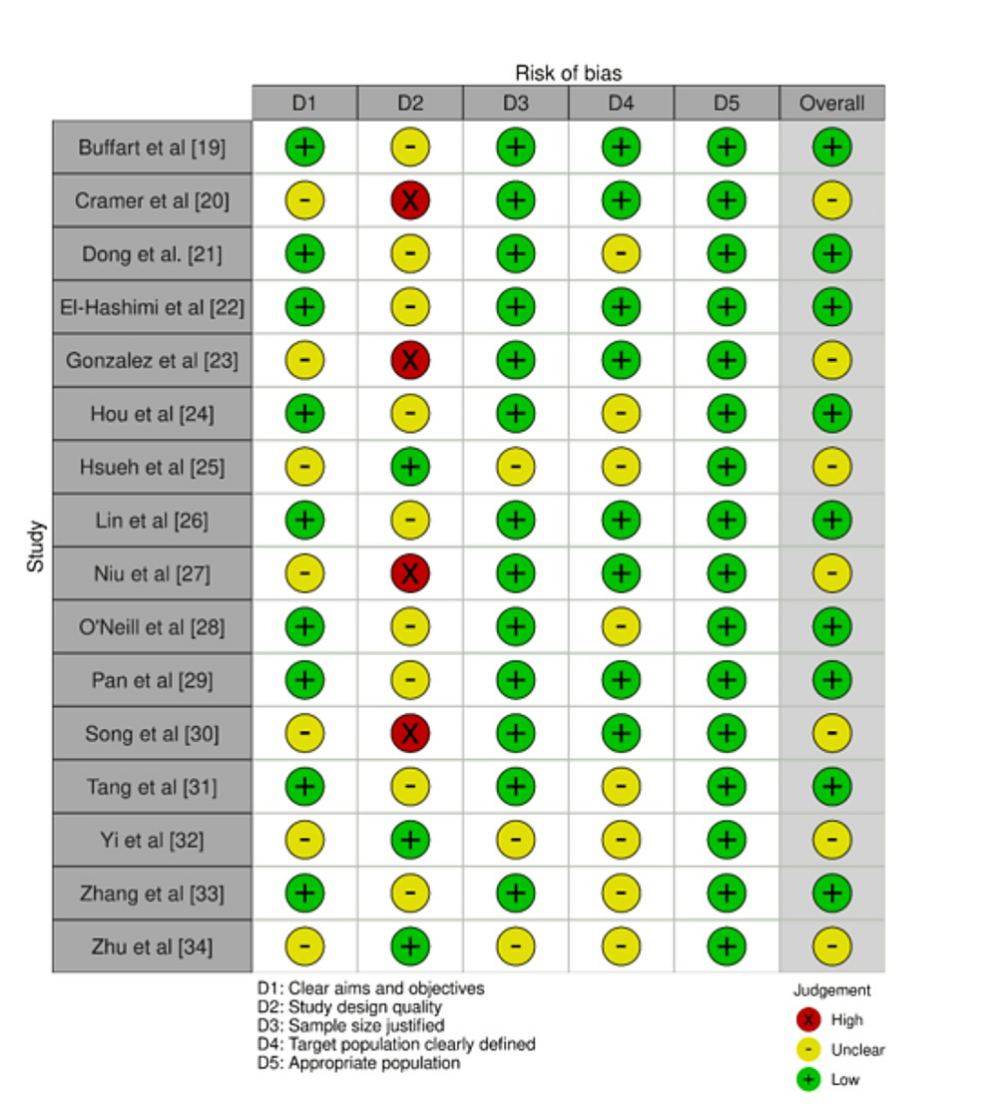
Bias assessment using AMSTAR-2 tool AMSTAR-2: A Measurement Tool to Assess Systematic Reviews-2

The bias assessment of the selected studies, evaluated using the AMSTAR-2 tool, indicated a general trend of low methodological quality across most studies. Specifically, studies by Buffart et al. [19], Dong et al. [21], El-Hashimi et al. [22], Hou et al. [24], Lin et al. [26], O'Neill et al. [28], Pan et al. [29], Tang et al. [31], and Zhang et al. [33] demonstrated low ratings across most criteria including unclear study design, unjustified sample sizes, and poorly defined target populations. These studies consistently showed an overall low-bias assessment (Figure 2).

Conversely, studies such as Cramer et al. [20], Gonzalez et al. [23], Niu et al. [27], and Song et al. [30] displayed high quality in study design. However, they were still marked by low scores in defining the target population and justifying sample sizes, leading to an overall unclear bias assessment. This suggests that while certain methodological aspects were adequately addressed, others were neglected, affecting the overall integrity of the findings.

The remaining studies, Hsueh et al. [25], Yi et al. [32], and Zhu et al. [34], also exhibited an overall unclear bias assessment due to mixed evaluations in various criteria. These studies were characterised by low clarity in aims and objectives and a general lack of detailed information regarding the study population and design. The variation in review quality has important implications for interpreting the overall findings of this umbrella review. High-quality reviews provide reliable evidence supporting the benefits of yoga in cancer care, such as significant reductions in anxiety, depression, and fatigue. Lower-quality reviews introduce uncertainty, particularly regarding the consistency and generalisability of these benefits. Methodological weaknesses in lower-quality reviews may lead to an overestimation of yoga's effectiveness, while high-quality reviews' rigorous methodologies lend greater credibility to their findings.

Pain Assessment

The studies reviewed present varied findings in evaluating pain levels among cancer patients engaging in yoga (Table 3). Buffart et al. [19] documented a substantial reduction in pain, particularly among breast cancer patients, highlighting the potential of yoga in managing pain within this specific subgroup. In contrast, Hsueh et al. [25] noted a decrease in pain severity, with a standardised mean difference (SMD) of -0.38 (95% CI: -0.74, -0.02), indicating a modest yet statistically significant improvement. Initially, Niu et al. [27] found no discernible impact on pain relief. However, subsequent sensitivity analysis revealed significant outcomes (SMD = -0.58), suggesting that yoga might be advantageous under certain circumstances or among specific patient subgroups.

Sleep Quality Assessment

Sleep quality, a critical component of overall well-being for cancer patients, showed varying improvements across studies. Cramer et al. [20] noted a significant short-term improvement in sleep quality with an SMD of -0.25 (95% CI: -0.40 to -0.09), indicating that yoga has a moderate beneficial effect on sleep disturbances. Zhu et al. [34] also observed significant improvements in sleep quality, specifically with meditation-focused yoga practised two to three times per week for six to eight weeks (SMD = -0.40, 95% CI: -0.71, -0.09, p = 0.01), suggesting that the type of yoga and its regularity might influence outcomes. Yi et al. [32] reported a significant improvement with an SMD of -0.34 (95% CI: -0.55, -0.12, p=0.003), reinforcing the notion that yoga can effectively improve sleep quality in cancer patients.

Fatigue

Fatigue is a prevalent and incapacitating symptom among cancer patients. The findings from the reviewed studies generally suggest that yoga can have a significant impact on this symptom. Cramer et al. [20] demonstrated that yoga resulted in a noteworthy short-term reduction in fatigue (SMD = -0.48, 95% CI: -0.75 to -0.20), although no medium-term effects were observed (SMD = -0.04, 95% CI: -0.36 to 0.29). Dong et al. [21] reported varying effects based on the type and timing of yoga, with the most pronounced reduction in post-treatment fatigue observed in supervised classes (SMD = -0.92). Similarly, Hsueh et al. [25] documented a significant decrease in fatigue levels with an SMD of -0.99 (95% CI: -1.56, -0.43), emphasising the potential effectiveness of yoga in managing cancer-related fatigue.

Exercise Capacity

The data on the exercise capacity of cancer patients involved in yoga practices were inconsistently documented in various studies. Only a limited number of studies, such as the one conducted by Zhu et al. [34], specifically evaluated parameters such as the Six-Minute Walk Test, yielding mixed results that showed slight improvement. This suggests further research, as exercise capacity constitutes a crucial aspect of cancer rehabilitation.

Cytokine Levels

The studies that reported on cytokine levels, such as Tang et al. [31], showed mixed outcomes, with significant decreases in inflammatory markers such as TNF-α and IL-6 in some cases. This suggests that yoga might influence immune function and inflammatory responses in cancer patients, although the results were variable. They indicate the need for more targeted studies to understand the relationship between yoga and cytokine levels comprehensively.

Anxiety and Depression

Multiple studies, such as those conducted by Hsueh et al. [25] and Yi et al. [32], have demonstrated substantial reductions in anxiety and depression among cancer patients who engage in yoga. For example, Hsueh et al. [25] reported moderate effect sizes for both depression (g = -0.553) and anxiety (g = -0.554), indicating a consistent and notable benefit of yoga in alleviating these psychological symptoms. The convergence of findings from various studies lends support to the incorporation of yoga as an effective intervention for addressing emotional distress in cancer care.

Quality of Life

Studies by Gonzalez et al. [23] and O'Neill et al. [28] frequently documented improvements in QOL. Gonzalez et al. [23] found significant enhancements in functional well-being, while O'Neill et al. [28] reported improvements in global health status. The standard mean differences (SMD) varied from modest to substantial, indicating that yoga may significantly contribute to improved overall well-being and QOL in cancer patients.

Discussion

The comprehensive review of meta-analyses indicates consistently favourable outcomes associated with yoga interventions in cancer care, particularly improvements in psychosocial well-being and QOL. Studies by Gonzalez et al., Hsueh et al., and Lin et al. demonstrate substantial reductions in anxiety, depression, and stress, highlighting yoga's effectiveness in managing psychological symptoms linked to cancer [23,25,26]. Research by Hou et al., O'Neill et al., and Pan et al. consistently observed enhancements in overall well-being, indicating a positive impact on QOL [24,28,29].

Regarding physical outcomes, studies by Dong et al., Zhu et al., and Song et al. reported significant improvements in fatigue and sleep quality, underscoring yoga's potential benefits in addressing physical symptoms associated with cancer treatments [21,34,30]. These findings suggest a broad consensus on yoga's favourable physical and psychological health effects.

However, differences in the duration and effectiveness of yoga interventions were noted. Cramer et al. pointed out that while yoga has significant short-term benefits, it did not consistently show superiority over other forms of exercise in the medium term [20]. This contrasts with studies such as Yi et al. and Tang et al., which reported significant and long-lasting effects on stress reduction and sleep quality [31,32].

The effectiveness of yoga also varied depending on the type of intervention and patient adherence. Dong et al. observed that effectiveness varied by the type and format of the yoga intervention, while Tang et al. noted variability based on patient adherence [21,31]. This variability underscores the contextual nature of yoga's effectiveness, suggesting that its benefits may be optimised by tailoring interventions to individual patient needs and ensuring consistent practice.

A strong consensus was evident in the context of psychosocial enhancements, with consistent reports of yoga's benefits. However, dissimilarities were most pronounced in studies examining yoga's long-term effects and comparative efficacy. For instance, Cramer et al. offered a critical assessment of yoga's medium-term efficacy compared to other exercises, presenting a nuanced perspective that challenges the notion of yoga's superiority in all aspects of cancer care [20].

Research indicates that the incorporation of yoga into cancer care has shown promise in mitigating adverse symptoms associated with the illness and its treatments. There is growing recognition of the benefits of yoga in oncology programmes, with evidence suggesting its potential to alleviate stress, anxiety, and depression [35-37]. Furthermore, there is an indication that yoga may impact cellular environments and immune function, potentially inhibiting tumour development and progression. However, rigorous studies are necessary to further explore yoga's therapeutic potential in cancer care [38-42].

Limitations of the Review

The study's limitations stem from methodological inconsistencies and the varied quality of the reviewed meta-analyses. Most studies demonstrated significant benefits of yoga in reducing psychological symptoms such as anxiety, depression, and stress and improvements in QOL and physical symptoms such as fatigue and sleep quality. However, the evidence highlighted significant variability in yoga interventions' persistence and comparative effectiveness. For instance, while yoga provided notable short-term benefits, it did not consistently outperform other forms of exercise in the medium term. The effectiveness of yoga also differed based on the type of intervention and patient adherence, indicating that the effectiveness of yoga may greatly depend on personalised interventions and patient commitment.

Recommendations

Healthcare providers should consider incorporating yoga as a complementary therapy for cancer patients, particularly for its psychosocial benefits. Integrating yoga programmes in cancer rehabilitation centres and offering them as part of patient care routines could elevate overall well-being. Yoga could provide a non-pharmacological option for physical benefits such as improved fatigue management and better sleep quality, reducing reliance on medication.

Tailoring yoga interventions to each patient's specific needs and capabilities is crucial, considering factors such as cancer type, treatment phase, and physical condition. Ensuring that yoga programmes are adaptable and accessible will likely increase patient adherence and maximise therapeutic outcomes.

Given that yoga does not consistently outperform other forms of exercise in the medium term, practitioners should maintain a balanced perspective, presenting yoga as one of several exercise options available to patients. Ongoing research and feedback mechanisms should be established to continuously assess and improve yoga interventions, involving regular follow-up with patients to gauge long-term outcomes and adjust programmes as necessary.

## Conclusions

The synthesis of findings from this umbrella review indicates that yoga is an effective supplementary therapy for cancer patients, significantly enhancing QOL and reducing psychosocial symptoms such as anxiety, depression, and stress. Additionally, improvements in physical symptoms such as fatigue and sleep disturbances further support overall well-being. However, the effectiveness of yoga interventions varies based on the type of yoga practised, the format of the intervention, and patient adherence, suggesting that benefits are not universally guaranteed and depend heavily on the specific structure and delivery of the yoga programme. Comparative analyses reveal that, while yoga provides substantial benefits, it is not superior to all other forms of supportive care. Instead, yoga should be considered a valuable component of a multidisciplinary approach to cancer care, complementing other therapeutic modalities. These findings highlight the need for personalised yoga interventions tailored to individual patient's unique needs and conditions to maximise therapeutic outcomes.

Furthermore, there are significant policy implications, including the development of guidelines and the allocation of funding for implementing yoga programmes in cancer care settings. Policymakers should recognise the value of holistic and integrative approaches to cancer treatment and support initiatives that facilitate the incorporation of yoga into clinical practice. Future research should prioritise high-quality, large-scale studies to confirm the benefits of yoga observed in this review. Investigations should focus on identifying the most beneficial elements of yoga practice for various patient demographics and cancer types and understanding the mechanisms through which yoga exerts its effects. This knowledge will enable the tailoring of yoga interventions to enhance therapeutic efficacy and provide robust evidence to support their inclusion in cancer care protocols.
